# Metabolic dysfunction-associated fatty liver disease: current therapeutic strategies

**DOI:** 10.3389/fnut.2024.1355732

**Published:** 2024-03-19

**Authors:** Khamis Al Hashmi, Rosaria Vincenza Giglio, Anca Pantea Stoian, Angelo Maria Patti, Khalid Al Waili, Khalid Al Rasadi, Marcello Ciaccio, Manfredi Rizzo

**Affiliations:** ^1^Department of Physiology, College of Medicine and Health Sciences, Sultan Qaboos University, Muscat, Oman; ^2^Department of Biomedicine, Neuroscience and Advanced Diagnostics, University of Palermo, Palermo, Italy; ^3^Department of Laboratory Medicine, University Hospital, Palermo, Italy; ^4^Department of Diabetes, Nutrition and Metabolic Diseases, Carol Davila University of Medicine and Pharmacy, Bucharest, Romania; ^5^Internal Medicine Unit, “Vittorio Emanuele II” Hospital, Castelvetrano, Italy; ^6^Department of Biochemistry, Sultan Qaboos University Hospital, Muscat, Oman; ^7^Department of Biochemistry, College of Medicine and Health Sciences, Sultan Qaboos University, Muscat, Oman; ^8^Medical Research Center, Sultan Qaboos University, Muscat, Oman; ^9^College of Medicine, Mohammed Bin Rashid University of Medicine and Health Sciences, Dubai, United Arab Emirates; ^10^Department of Health Promotion Sciences, Maternal and Infant Care, Internal Medicine and Medical Specialties (PROMISE), University of Palermo, Palermo, Italy

**Keywords:** Metabolic Associated Fatty Liver Disease (MAFLD), Nonalcoholic Fatty Liver Disease (NAFLD), Cardiovascular Disease (CVD), nutraceuticals, innovative therapies

## Abstract

The definition of “Metabolic Associated Fatty Liver Disease – MAFLD” has replaced the previous definition of Nonalcoholic Fatty Liver Disease (NAFLD), because cardiometabolic criteria have been added for the prevention of cardiological risk in these patients. This definition leads to an in-depth study of the bidirectional relationships between hepatic steatosis, Type 2 Diabetes Mellitus (T2DM), Cardiovascular Disease (CVD) and/or their complications. Lifestyle modification, which includes correct nutrition combined with regular physical activity, represents the therapeutic cornerstone of MAFLD. When therapy is required, there is not clear accord on how to proceed in an optimal way with nutraceutical or pharmacological therapy. Numerous studies have attempted to identify nutraceuticals with a significant benefit on metabolic alterations and which contribute to the improvement of hepatic steatosis. Several evidences are supporting the use of silymarin, berberine, curcumin, *Nigella sativa*, *Ascophyllum nodosum*, and *Fucus vesiculosus*, vitamin E, coenzyme Q10 and Omega-3. However, more evidence regarding the long-term efficacy and safety of these compounds are required. There is numerous evidence that highlights the use of therapies such as incretins or the use of Proprotein Convertase Subtilisin/Kexin type 9 (PCSK9) inhibitors or other similar therapies which, by assisting existing therapies for pathologies such as diabetes, hypertension, insulin resistance, have given a breakthrough in prevention and the reduction of cardiometabolic risk. This review gave an overview of the current therapeutic strategies that are expected to aid in the treatment and prevention of MAFLD.

## 1 Introduction

**Figure 1:**
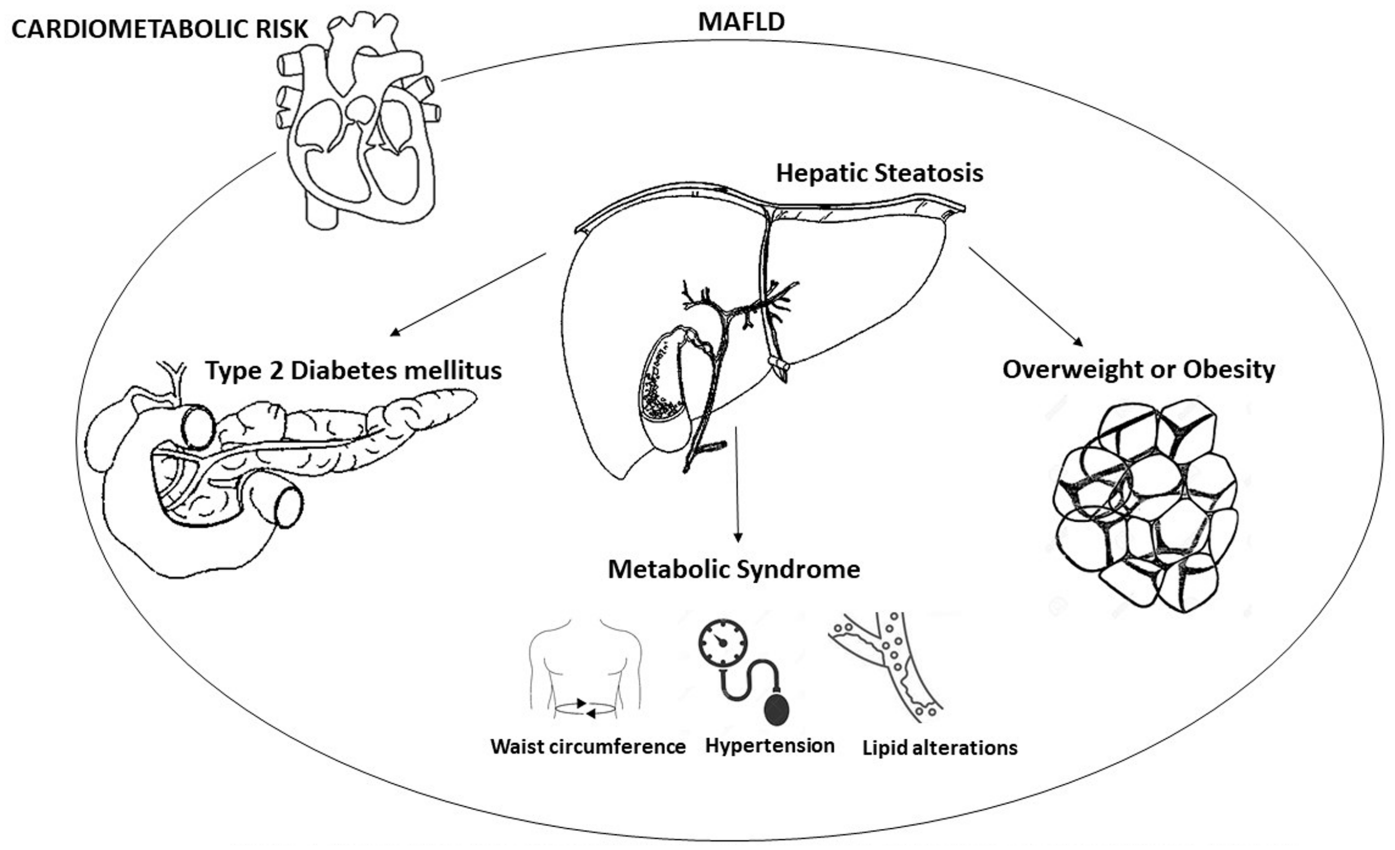
Metabolic factors involved in metabolic dysfunction-associated fatty liver disease (MAFLD).

In recent years, disapprovals took place on the traditional naming of Nonalcoholic Fatty Liver Disease (NAFLD) which excludes excessive alcohol assumption and the absence of many chronic liver diseases. Therefore, the knowledge on the metabolic alterations of the disease and its high risk of cardio-metabolic complications have raised the necessity to find a new denomination that introduces valid criteria that describe the underlying pathophysiological mechanisms. The new definition “Metabolic Associated Fatty Liver Disease – MAFLD” identifies a condition of hepatic steatosis associated with metabolic alterations that are defined by means of clear and easy-to-apply diagnostic criteria (1) and which underlines the importance of obesity, diabetes, and Metabolic Syndrome (MetS) in the pathogenesis of fat liver (Figure 1) (2). Numerous evidences suggest a strong association between an increase in cardiovascular risk and NAFLD. The Adolescent Brain Cognitive Development (ABCD) study shows that patients affected by fatty liver disease, matched to those who are free of the disease and after managing obesity, have higher Atherosclerotic Cardiovascular Disease (ASCVD) score values and express a higher 10-year risk of atherosclerotic cardiovascular disease events (3). Other clinical trials underline that fat liver is an independent risk factor both for enhancement carotid intima-media thickness (4) and for the presence of echocardiographic signs of diastolic cardiac dysfunction (5).

The association between NAFLD and cardiovascular risk is increasingly documented by studies that highlight ultrasound evaluations relating to the presence of carotid plaques after at least 2 years and the presence of fatty liver at the time of baseline evaluation as a factor of independent risk for the appearance of carotid atherosclerotic plaques (6).

MAFLD tends to include more than NAFLD patients with metabolic alterations, and therefore with greater cardiovascular risk, older patients, subjects with advanced fibrosis, and patients with other associated liver diseases (the diagnosis is no longer one of exclusion). The internist and the diabetologist become a key figure in the diagnosis and initial care of subject affected by metabolic hepatic pathology. The criteria for the MAFLD diagnosis are important for the appropriate use of innovative therapies and nutraceutical supplements.

Therefore, tailored therapies are needed for the corollary cardiovascular risk lower factors in subject affected by MAFLD and to improve liver function.

### 1.1 Aim of the review

There is no consensus on the best therapeutic approach to manage patients with MAFLD. The aim of this review is to provide an overview of the current therapeutic strategies, whether pharmacological or otherwise, for the treatment and prevention of MAFLD.

## 2 Metabolic dysfunction-associated fatty liver disease (MAFLD)

Currently, NAFLD is the most frequent hepatic alteration; NAFLD is considered as the presence of fat in liver echography and/or biopsy in the absence of one more hepatic injury (alcohol, hepatotoxic drugs, toxins, viral infections, genetic liver disease) (7).

However, the term NAFLD refers only to liver disease unrelated to alcohol and not to alterations on metabolism and correlated cardiometabolic risk, for that reason, specialists in scientific societies have taken action to modify and improve this definition. The term MAFLD was proposed in 2020 and is diagnosed on the presence of fatty liver disease (verified by liver echography and/or biopsy) and the presence of one of the following comorbidities: Type 2 Diabetes Mellitus (T2DM), obesity and metabolic dysfunction (1).

The prevalence of this systemic disease is growing and in the future it could be the main cause of chronic hepatic condition (8).

The prevalence of obesity is implicated in the genesis of MAFLD, and the liver is often involved in obesity because the major metabolic processes linked to glucose and lipids take place in this organ. However, the metabolic dysfunctions do not only occur in the liver but also in other body area; in fact, Chronic Kidney Disease (CKD), osteoporosis, Obstructive Sleep Apnea Syndrome (OSAS), endocrine disorders, depression, cognitive impairment and cardiovascular consequences happen in these patients (9).

There is not accord on how the degree of fatty liver influence atherosclerosis. The association between the severity of fatty liver disease and atherosclerosis may be explained in part by severe lipotoxicity, inflammation, and marked hepatic insulin resistance (10). However, NAFLD/MAFLD still needs to receive enough interest in the community of cardiologists (11). The relationship between MAFLD and Cardiovascular Diseases (CVD)/Subclinical Carotid Atherosclerosis (SCA) is generally assigned to presence of shared risk factors (lipid abnormalities and obesity) (12). MAFLD focuses in the systemic metabolic conditions accompanying steatotic hepatic pathology.

Thus, the relationship between the development of MAFLD and SCA needs to be further explored.

### 2.1 Metabolic profile of MAFLD

MAFLD is defined as the presence of fatty liver disease associated with at least two metabolic alterations among (13):

- Waist circumference ≥ 102/88 cm in Caucasian men/women or ≥ 90/80 cm in Asian men/women;
- Blood pressure ≥ 130/85 mmHg or antihypertensive drugs;
- Plasma Triglycerides (TG) ≥ 150 mg/dL or TG lowering drugs;
- Plasma high-density lipoprotein cholesterol (HDL-C) < 40 mg/dL for men and < 50 mg/dL for women or lipid-lowering drugs;
- Fasting plasma glucose levels between 100 and 125 mg/dL or 2 h post-load;
- Glucose levels between 140–199 mg/dL or glycosylated hemoglobin (HbA1c) between 5.7–6.4%;
- Homeostasis model assessment (HOMA) with insulin resistance score ≥ 2.5;
- High-sensitivity C-reactive protein levels >2 mg/L.

Hepatic steatosis is diagnosed by ultrasound and the hepatic parenchyma appears brightness (14). The presence of steatosis is not always an indication of MAFLD. The possible alternative causes of steatosis (drugs, celiac disease, prolonged fasting, severe weight loss, lipid metabolism disorders) must always be sought in the absence of the clinical criteria of MAFLD.

The pathophysiology of MAFLD is complicated and heterogeneous. The “two-hit hypothesis” proposed by some colleagues predicts that the first hit causes fat accumulation in hepatocytes and the second hit causes oxidative stress, increasing inflammation and leading to fibrosis (15). The second hypothesis instead proposed that MAFLD develops when the synthesis of triglycerides in the liver exceeds the catabolism of non-esterified fats and depends on the oxidation in the mitochondria and the export of the same triglycerides in Very Low-Density Lipoproteins (VLDL) (“multiple hit hypothesis”) (16). In patients with MAFLD, the expression of Acetyl-Coenzyme-A Carboxylase 1 (ACC1), an enzyme in *de novo* lipogenesis, was decreased and acetyl-coenzyme-A was shown to be converted to malonyl-CoA. Accumulation of malonyl-CoA inhibits Carnitine Palmitoyl Transferase (CPT)-1, which transports fatty acids into the mitochondria and decreases β-oxidation. Fatty acid synthase (FAS) induces the conversion of malonyl-CoA to palmitic acid, and its expression is reduced in NASH patients (17), but the mechanism leading to the decrease in triglyceride synthesis appears to be the accumulation of harmful free fats.

MAFLD has been associated with MetS and it has been considered the liver expression of the MetS. MAFLD may facilitate the development of components of the metabolic syndrome, which may in turn predispose to MAFLD (14).

Patients with diabetes have a prevalence of MAFLD two to three times higher than patients without. The improper deposit of fat in the liver causes alterations in energy metabolism and the inflammatory state, generating insulin resistance. Due to this chronic hyperinsulinemia, patients with diabetes tend to accumulate more fat in the liver. In these patients the severity, morbidity, progression, and liver-related mortality associated with MAFLD are much higher (16, 17).

A distinction is no longer made between steatosis and steatohepatitis (therefore, the diagnostic concept of Metabolic dysfunction-Associated Steatohepatitis (MASH) does not exist), but the disease activity as well as the fibrosis are evaluated as a continuum based on the data of non-invasive methods (Table 1) or liver biopsy (NAFLD Activity Score and degree of fibrosis).

**Table 1:** Clinical-laboratory indexes for monitoring the steatosis activity and the fibrosis.

Clinical-laboratory indexes
AST/platelet ratio index (APRI)
BARD score
Enhanced Liver Fibrosis (ELF) score
Fatty Liver Index (FLI)
Fibrosis-4 (FIB-4) index
Fibrotest
Hepatic Steatosis Index (HSI)
HepaScore
Nonalcoholic Fatty Liver Disease (NAFLD) Fibrometer
Nonalcoholic Fatty Liver Disease (NAFLD) Fibrosis score
Nonalcoholic Fatty Liver Disease (NAFLD) Liver Fat Score
SteatoTest

Laboratory tests support the MAFLD diagnosis and allow us to evaluate the conditions associated with hepatic steatosis evolution. Generally, liver fibrosis biomarkers reflect matrix turnover but not the extent of extracellular matrix deposition. None of the biomarkers available today are specific to fibrosis in the liver, and inflammatory and oxidative states in other sites can contribute to increasing circulating levels.

The indirect markers, which reflect alterations in liver function such as ASpartate aminoTransferase (AST), ALanine aminoTransferase (ALT), platelet count, Gamma-Glutamil Transferas (GGT), total bilirubin, alpha 2-macroglobulin or alpha 2-globulin (mainly haptoglobin), individually provide rather limited clinical information regarding the presence or absence of fibrosis. Hence, scores that consider multiple biomarkers combined in various ways which increase diagnostic accuracy (18) have been proposed (Table 1).

Direct markers which instead reflect liver fibrosis contain biomarkers of collagen synthesis or degradation, extracellular matrix glycoproteins, proteoglycans, and glycosaminoglycans (PIIINP: amino-terminal Propeptide of type III Procollagen; TIMP-1: Tissue Inhibitor of Metalloproteinase; TNF: Tumor Necrosis Factor; MMP: Matrix MetalloProteinase) must be consider. Furthermore, in the pathophysiological mechanisms involved in MAFLD, pro-inflammatory molecules such as Transforming Growth Factor beta-1 (TGF-β1), Insulin-Like Growth Factor (IGF-1) and endothelin-1 and inflammatory mediators such as C-Reactive Protein (CRP), Interleukin (IL)-6, and pro-coagulant factors such as fibrinogen, factor VIII and plasminogen activator inhibitor-1 (19), which also determine insulin resistance (20) must also be considered.

There are different pathophysiological mechanisms underlying the link between MAFLD and atherosclerosis (21). In conditions of insulin resistance, the action of lipases leads to an abnormal flow of fatty acids and the production of chylomicrons (CM) at the intestinal level and VLDL to the liver. Hyperinsulinemia leads to increase fatty acid esterification and inhibit beta-oxidation which determining the triglycerides formation in liver. Dysmetabolic subjects have systematically more active processes of oxidative stress, increase levels of glucose in circulating and increase in blood lipoproteins, leading to foam cell formation and atherosclerotic disease (22).

### 2.2 Cardiovascular risk in MAFLD

The arterial stiffness and endothelial dysfunction present in MAFLD determine the increase in mortality for cerebro-cardiovascular events. The cerebral hemodynamics modifications are detected by transcranial Doppler. This instrumental investigation allows to measure alterations such as the blood flow velocity of the middle cerebral artery, the Pulsatility Index (PI) and the Resistance Index (RI), markers of cerebrovascular vasoconstriction and the Index of Respiratory Retention (RRI).

Subclinical atherosclerosis is significantly correlated with an elevated risk of cardiovascular disease. Carotid Intima-Media Thickness (CIMT) and carotid plaque are surrogate markers for Acute Coronary Syndrome (ACS) risk (23). Through B-mode ultrasound, it is possible to examine the carotid artery, observing the bilateral parts of the internal, external, common, and bifurcations sites (24). High CIMT is defined as CIMT >1.1 mm and identified as carotid plaque CIMT >1.5 mm. ACS is diagnosed with carotid artery plaque or increased CIMT (24).

Subclinical atherosclerosis is closely related to NAFLD as demonstrated in previously conducted studies (25). This close association also applies to MAFLD as reflected in CIMT alterations in these patients (26). The impact of carotid atherosclerosis in MAFLD are most evident in younger subjects or those with severe fatty liver disease. NAFLD has insulin resistance as its pathophysiological mechanism and is regarded a manifestation of cardiometabolic alteration (27). Unfortunately, however, NAFLD is underestimated as an independent risk factor for ASCVD (28) and better attention should be paid to the diagnosis, monitoring, and management of these patients.

Several studies and meta-analyses have established the correlation between NAFLD and the onset of ACS. Surrogate markers of cardiovascular disease such as the presence of carotid plaque, carotid intima-media thickness, brachial artery vasodilatory responsiveness and coronary artery calcification score have been associated to NAFLD (29). MAFLD has been shown to identify high-risk subjects for fibrosis, metabolic dysfunction, and chronic kidney disease (30).

## 3 Current treatment of MAFLD

To date, there is no consensus on the best therapeutic approach, whether pharmacological or otherwise, for patients with MAFLD.

The therapeutic goals in patients with MAFLD are to reduce steatosis, chronic inflammation, and fibrosis and control the main cardio-metabolic risk factors. In this way, the reduction of hepatic and extrahepatic complications and cardiovascular mortality is hypothesized. According to clinical practice guidelines, optimal therapeutic strategies to halt the progression and development of MAFLD and CVD include lifestyle modification, smoking cessation, weight reduction, dietary intervention, and exercise (31). Smoking cessation is essential for the prevention of the primary causes of MAFLD and CVD (31). In overweight and obese patients with MAFLD, a weight loss of 7–10% is desirable to attain a reduction in hepatic steatosis and vascular and metabolic complications (31). The recommendations for both the treatment of MAFLD and the prevention of cardiovascular diseases envisage a low-carbohydrate, ketogenic, low-fat, high-protein, Mediterranean diet, which causes a reduction in dyslipidemia, fatty liver disease and of its associated comorbidities (31). High-intensity interval exercise, which has been shown to improve plasma lipid levels and insulin resistance as well as CVD risk factors, such as plasma levels of triglyceride-rich VLDL1 particles and LDL cholesterol, should be recommended in combination with proper diet (32).

In addition to modifying lifestyle, which must gradually leads to weight loss and therefore reduction of visceral fat, in recent years, attention has focused on the use of nutraceuticals and/or innovative therapies with proven improving effects on liver enzymes, hepatic steatosis, the reduction of insulin resistance, the lipid profile and that can in a more specific and tailor reduce the possibility of the onset of a cardiovascular event in patients affected by this condition. There is no pharmacological therapy with special indication for MAFLD. All therapies are off-label.

### 3.1 Nutraceutical supplements

The use of nutraceuticals for the management of NAFLD is a proposition that should not be underestimated. Nutraceuticals alone or in combination with diet and lifestyle modifications promoting weight loss and reducing insulin resistance (33). The use of nutraceuticals in terms of therapeutic intervention in the presence of NAFLD is evidence-based. However, partly due to the relative limitations of studies in this area compared to marketing studies on new drugs and partly due relatively small sample size with a short interval of follow-up, studies are basing their conclusions on surrogate endpoints, rather than purely clinical outcomes (34). The nutraceuticals considered are those bring a significant advantage on cardio metabolic risk, that contribute to the improvement of hepatic steatosis and act by improving metabolic factors. Some of these have rather low bioavailability, which could compromise their effectiveness. Individual genetic composition influences how nutraceuticals are assimilated, stored, and excreted and represents an individualized approach to disease.

Nutraceuticals have effects on health through different actions (inflammation, glycemia and insulinemia, LDL-C, hypertension). In addition, they have complementary actions and can have effects on different biomolecular targets (35).

*Silymarin* is an antioxidant composed of seven flavonolignans (silybin A, silybin B, isosilybin A, isosilybin B, silychristin, iso-silychristin, and silydianin) and one flavonoid (taxifolin). It represents one of the highly used natural compounds in the management of liver disorders for its anti-inflammatory, antioxidant, antifibrotic and insulin-sensitive properties. In preclinical studies performed in mice models, it has been shown that a nutraceutical containing silymarin together with chlorogenic acid, guggul, curcumin and inulin was able to prevent NAFLD and atherosclerosis (36). Also, in patients with NAFLD, the combination of silybin, phosphatidylcholine and vitamin E, administered for 12 months, reduced transaminases, GGT and the degree of steatosis (37). A meta-analysis of eight RCTs showed a significant decrease in aspartate aminotransferase (AST) and alanine aminotransferase (ALT) levels. Several other studies confirmed a reduction in HOMA-IR, glycemia and insulinemia in patients affected by NAFLD (38).

*Berberine* is a quaternary ammonium salt of the isoquinoline alkaloid group known for its lipid-lowering and insulin-sensitizing activity (39). Treatment with 0.5 g of berberine resulted on a significant improvement in the lipid profile and a significant reduction in body weight, HOMA-IR, and hepatic steatosis (40). Furthermore, the coadministration of berberine and silymarin has been shown to be associated with several significant ameliorations in both the lipid and glucose profiles, suggesting that cardiometabolic and health level can be promoted with the potential use of this nutraceutical combination (41).

*Curcumin*, extracted from *Curcuma longa*, is an insulin-sensitizing nutraceutical that significantly reduces hepatic steatosis (42). Supplementation with curcumin for 8 weeks resulted in a significant improvement in insulinemia, HOMA-IR, waist circumference, blood pressure, TG, HDL-C, hepatic transaminases, GGT and hepatic steatosis in subjects with prediabetes (43).

*Nigella sativa* belongs to the Ranunculaceae family, whose antioxidant and anti-inflammatory outcomes are attributable to thymoquinone and is used for the treatment of liver diseases (44). Supplementation with *Nigella sativa* significantly improves transaminases, fasting blood sugar, lipid profile, high-sensitivity C-reactive protein and the degree of hepatic steatosis (45).

The combination of *Ascophyllum nodosum* and *Fucus vesiculosus* which slows the intestinal absorption of cholesterol by increasing intestinal viscosity and reduces the absorption of sugars by inhibiting the enzymes α-amylase and α-glucosidase, significantly reduces insulinemia, HOMA-IR, blood glucose, and also waist circumference. In addition, it significantly increases plasma HDL-C levels after 6 months of therapy (46).

*Coenzyme Q10* has anti-inflammatory properties taken into consideration in managing hepatic steatosis and metabolic alterations (47). Coenzyme Q10 regulates adipokine levels and decreases oxidative stress in patients with metabolic syndrome. The intake of 100 mg/day of coenzyme Q10 for 3 weeks led to a reduction in transaminases and GGT, an improvement in the adiponectin/leptin ratio and better glucose control (48). Coenzyme Q10 has a high safety profile without relevant pharmacological interactions. However, its relatively low bioavailability raised limitation concerns for its use.

*Polyunsaturated fatty acids* of the omega-3 series could play a role in the treatment of metabolic and hepatic disorders characterizing MAFLD (49). Long-term supplementation with omega-3 is associated with a significant improvement in AST, ALT, and the degree of hepatic steatosis (50), TG, HOMA-IR and glycemia (51).

The synergistic nutraceutical combination therapy prescribed for MAFLD could benefit a series of diseases perhaps unknown to the patient and indirectly treated can help in the prevention of cardiovascular risk factors. Silymarin, polyunsaturated fatty acids of the ω-3 series, coenzyme Q10, berberine and curcumin possess hepatoprotective activity and exert a favorable action on the CV system. Traditional Chinese herbal combinations such as Artemisia capillaris (Thunb), *Gardenia jasminoides* (Ellis), and *Rheum palmatum* (L) exert anti-NAFLD effects (33).

The consumption of chlorogenic acid and its derivatives and luteolin and its derivatives by individuals with MetS with a follow-up period of 6 months shown a significant improvement in body weight (*p* < 0.001), waist circumference (*p* = 0.003), HbA1c (*p* < 0.001), plasma lipids (*p* < 0.001 for Tchol. LDL and TG), Fatty Liver Index (FLI), a surrogate marker of fatty liver disease (*p* < 0.001), liver transaminases, flow-mediated dilatation (*p* < 0.001), and carotid intima-media thickness (*p* < 0.001), regardless of the degree of fatty liver disease (52). The effect of a supplement containing *Curcuma longa*, silymarin, guggul, chlorogenic acid, and inulin was evaluated in patients with MetS for 4 months. There were significant reductions in body weight (*p* < 0.0001), Body Mass Index (BMI) (*p* = 0.001), waist circumference (*p* = 0.0004), fasting glucose (*p* = 0.014), and total cholesterol (*p* = 0.03) (53).

### 3.2 Pharmacological treatment

Drug therapies for MAFLD management tend to decrease fat accumulation in the liver, activate metabolic pathways, and improve liver damage.

Acetylsalicylic acid (aspirin) is recommended in an established atherosclerotic disease, reduces liver fibrosis, and prevents cardiovascular events (31). Statins are key elements in the pharmacological modification of cardiovascular risk and are recommended for patients with MAFLD and NASH (54). In subjects with elevated transaminases, atorvastatin reduced cardiovascular risk due to both cardiological and hepatological benefits (31). The use of ezetimibe is tolerable and effective for the prevention of CVD in MAFLD. It reduces hepatic lipid synthesis and improves liver histology (55).

Bempedoic acid, an inhibitor of ATP citrate lyase (an enzyme involved in the synthesis of cholesterol upstream of HMGCoA reductase), reduces LDL cholesterol by 28% in monotherapy and in combination with statins and ezetimibe (56).

Proprotein Convertase Subtilisin/Kexin type 9 (PCSK9) influences muscle and hepatic lipid accumulation and contribute to the pathogenesis and progression of MAFLD. No liver-related negative signals were reported in the Fourier (Evolocumab) and Odyssey (Alirocumab) studies, and it appears to be safe in the patients with liver disease (31). A beneficial activity is exerted by Pemafibrate, which is much more selective for PPAR alpha than PPAR gamma or PPAR delta (56). Pemafibrate improved macrophage accumulation, and ballooning degeneration of hepatocytes without a noticeable change in TG accumulation in the liver (57). It also improved markers of liver inflammation, function and fibrosis (58).

Obeticholic acid (OCA), a semisynthetic variant of the naturally occurring bile acid chenodeoxycholic acid, increased insulin sensitivity and decreased markers of liver fibrosis in NAFLD patients with T2DM (59).

The presence of MAFLD in T2DM raise the risk of microvascular complications (especially diabetic nephropathy), increases cardiovascular risk, and has a three times greater risk of presenting itself in the form of advanced fibrosis or cirrhosis or hepatocellular carcinoma. The hypoglycemic drugs used for the treatment of diabetes, a disease often associated with the onset of MAFLD, have been used for a long time in the treatment of MAFLD. Metformin used in MAFLD patients with T2DM had a greater benefit on improving aminotransferase levels than vitamin E treatment (60). In MAFLD, metformin directly reduced fat deposition and inhibited inflammation in the liver by enhancing phosphorylation of hepatic 5′ adenosine monophosphate-activated protein kinase (AMPK) and ACC and reduced lipogenic enzymes and proinflammatory cytokines (61). Sodium-glucose cotransporter-2 (SGLT2) inhibitors improve liver enzymes AST and ALT and liver fat accumulation (62).

SGLT2 inhibitors reduce body fluids and body weight and may be recommended in obese subjects with MAFLD (63). Treatment with canagliflozin for 20 weeks delayed the onset of NASH and caused reduction in liver enzymes [ALT, AST, Alkaline Phosphatase (ALP), and GGT] and body weight, and increased bilirubin (64). Empagliflozin reduces liver fat content and improves hepatic steatosis and fibrosis, decreases AST and ALT in MAFLD patients with or without T2DM (64). Furthermore, it decreased the expression of hepatic inflammatory genes such as TNF-α, interleukin-6 and Monocyte Chemoattractant Protein-1 (MCP-1) in NASH and in combination with linagliptin, a DPP-4 inhibitor, it reduces mRNA expression for genes associated with fatty acid synthesis, collagen deposition and expression of Alpha Smooth Muscle Actin (αSMA), which is an indicator of fibrosis (64). Empagliflozin reduces insulin resistance and attenuates inflammasome and triglyceride NLR family pyrin domain containing 3 (NLRP-3) activation in the liver (64). Empagliflozin has shown a beneficial effect on steatosis, ballooning, and fibrosis (65). Ipragliflozin reduced thiobarbituric acid reactive substances and carbonyl and inflammatory protein markers in NASH (66).

Another class of drugs that benefit patients with MAFLD are the Glucagon-Like Peptide-1 (GLP-1) Receptor agonists (GLP-1RAs). GLP-1RAs delay the progression of MAFLD by inhibiting inflammation, insulin resistance, oxidative stress, enzymes participated in hepatic lipogenesis, and stimulating the autophagy/mitophagy pathway, as well as enzymes responsible for β-oxidation (67–71). There is a downregulation of GLP-1 receptors in MAFLD (72): GLP-1 analogs inhibit MAFLD by inhibiting the NLRP3 inflammasome through potentiation of autophagic/mitophagic pathways, increasing antioxidant defense in the liver and inhibiting macrophage recruitment and activation (73). They improve insulin sensitivity by reducing JNK phosphorylation and enhancing PPARγ expression and activity (74). These drugs decrease liver enzymes, body weight, and liver content. Exenatide treatment reduced fatty liver disease associated with decreased body weight, visceral fat, and fasting glucose levels (75). In the Liraglutide Efficacy and Action in NASH (LEAN) study, 39% of patients treated with liraglutide showed resolution of steatohepatitis on liver biopsy with improvements in blood glucose, HbA1c, GGT, and HDL, along with a weight loss of approximately 5 kg (76). Use of Liraglutide in patients with T2DM and NAFLD reduced CIMT, a surrogate marker of atherosclerosis, independently of glucometabolic changes (77). The use of semaglutide in patients with diabetes reduces hepatic fibrosis and steatosis parameters and the anthropometric, hepatic, and glycemic indices, as well as plasma lipids independent to the variations in CIMT and HbA1c (78).

Dipeptidyl Peptidase-4 (DPP-4) inhibitors work by impeding the activities of (DPP-4) to increase incretin levels and decrease glucagon release by increasing exocytosis of the insulin and fatty acid oxidation in the liver, decreasing gastric emptying, and lowering hepatic glucose production (79). Sitagliptin, a glyptin-based drug, has been found to cause reduction in liver enzymes, body weight, and hepatocyte swelling in patients with diabetes and NASH (80), but randomized clinical trials of this class of DPP-4 inhibitors in patients with NASH are needed.

## 4 Conclusion

**Figure 2:**
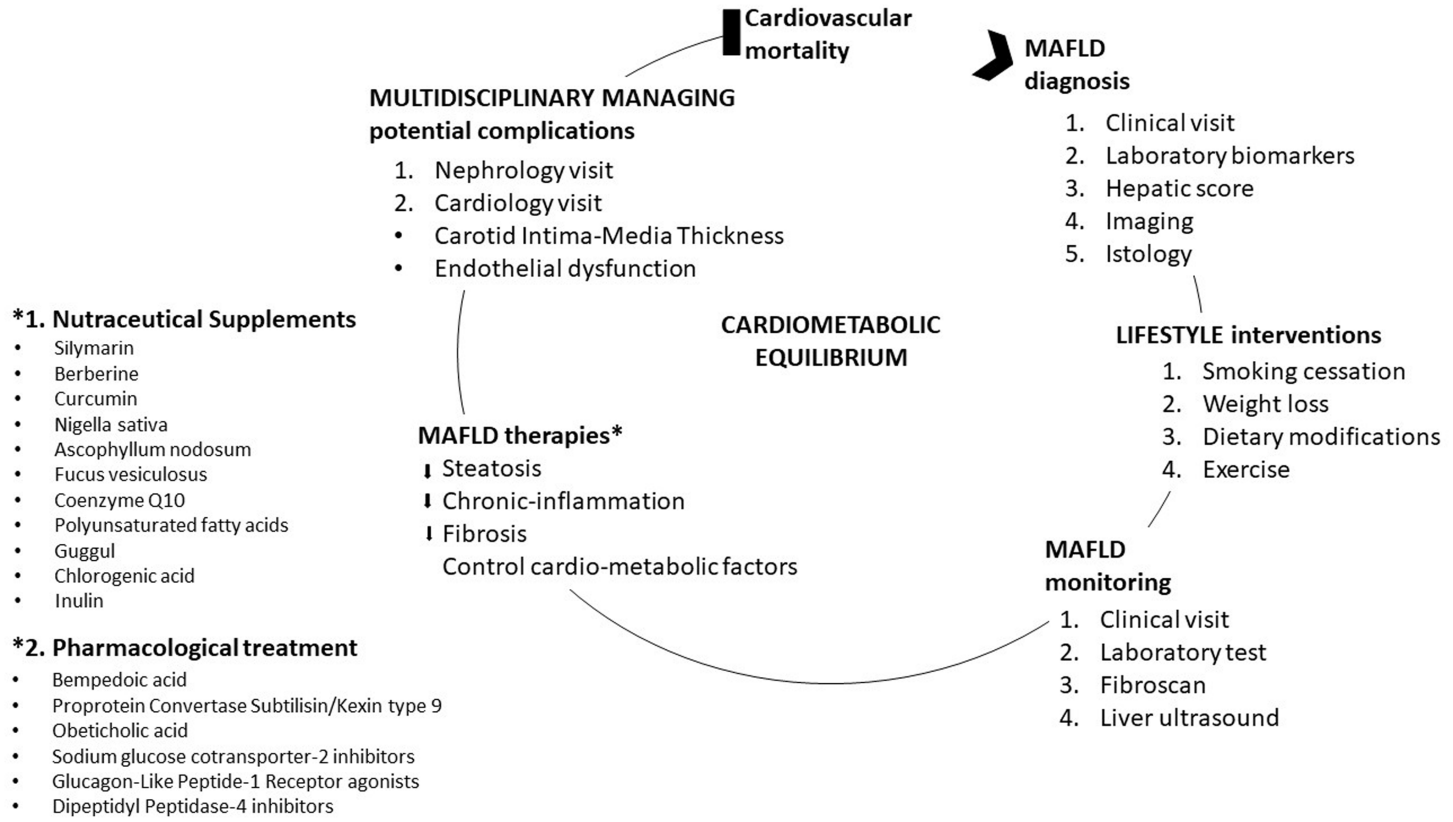
Management of metabolic dysfunction-associated fatty liver disease (MAFLD).

MAFLD has a very important impact on the healthcare system. MAFLD is a fairly complex pathology, a condition for which strict metabolic control a tailored therapy that can also be efficient in the prevention of cardiovascular complications as well as in the treatment of the pathologies present in its corollary as T2DM is required (Figure 2). The replacement of the term “non-alcoholic” with the term “metabolic” of fatty liver disease brings with it series of psychological mechanisms that favor greater awareness and attention to the disease on the part of patients, doctors, and the pharmaceutical industry.

There are no specific therapies for MAFLD. It is clear that drugs used for the treatment of diabetes, obesity, insulin resistance, hypercholesterolemia and hypertension could be proposed for the treatment of MAFLD and studies on the mechanisms that regulate lipid accumulation in the liver may lead to promising future therapies. Incretin therapies have an anti-inflammatory, lipid-lowering action, and act directly on the atheromatous plaque, blocking its progression. In addition, they act on body weight directly on the metabolism of adipocytes and have a direct effect on hepatic steatosis. The PCSK9 inhibitor acts instead by modulating both the internalization of the LDL receptor at the liver level, but also by improving hepatic steatosis. All this leads to a reduction in serum and endothelial LDL with a reduction in the risk of plaque formation and progression. Innovative therapies have proven to be effective in managing patients with diabetes, obesity, insulin resistance and hypercholesterolemia, but also in patients with MAFLD (56–81).

The evidence currently available regarding the use of nutraceuticals suggests hepatoprotective effects and positive outcomes on the metabolic front (33–53, 82, 83). Further studies are required to confirm observations and optimize nutraceutical treatment for MAFLD and to ensure their long-term efficacy and safety. In addition, it is wise to emphasize that dietary modification represents a very important lifestyle changes that over the years has been associated with lower cardiometabolic risk. The burden of cardiometabolic diseases is lowered by appropriate diet rich in proteins of animal and vegetable origin and poor in meat (84). The Mediterranean diet is still proposed by international guidelines as the treatment of choice (85, 86). To successfully alleviate MAFLD and associated comorbidities, new molecular markers should be identified and use as specific targets for the treatment of this pathology with high cardio-metabolic risk (87, 88).

## Author contributions

KAH: Supervision, Writing – original draft. RVG: Conceptualization, Supervision, Writing – original draft. APS: Supervision, Writing – review & editing. AMP: Conceptualization, Supervision, Writing – original draft. KAW: Writing – review & editing. KAR: Writing – review & editing. MC: Conceptualization, Supervision, Writing – review & editing. MR: Conceptualization, Supervision, Writing – review & editing.
